# EHD1 confers resistance to cisplatin in non-small cell lung cancer by regulating intracellular cisplatin concentrations

**DOI:** 10.1186/s12885-016-2527-3

**Published:** 2016-07-13

**Authors:** Jing Gao, Qingwei Meng, Yanbin Zhao, Xuesong Chen, Li Cai

**Affiliations:** The Fourth Department of Medicine Oncology, Harbin Medical University Cancer Hospital, 150 Haping Road, Harbin, 150040 China

**Keywords:** NSCLC, CDDP-resistant, EHD1, Intracellular concentrations

## Abstract

**Background:**

Non-small cell lung cancer (NSCLC) is one of the most aggressive types of cancer. However, resistance to cisplatin (CDDP) remains a major challenge in NSCLC treatment. The purpose of this study was to investigate the ability of EHD1 [Eps15 homology (EH) domain - containing protein 1] to confer CDDP resistance in NSCLC cells and to investigate mechanisms of this resistance.

**Methods:**

The associations between EHD1 expression in NSCLC specimens and clinicopathological features, including prognosis, were assessed by immunohistochemistry (IHC). Using DNA microarrays, we performed a genome-wide analysis of cisplatin-resistant NSCLC cells to identify the involvement of the EHD1 gene in this resistance. We overexpressed and knocked down EHD1 in cell lines to investigate the effect of this gene on proliferation and apoptosis. A quantitative analytical method for assessing CDDP in cells was developed. High-performance liquid chromatography was used to measure the concentration of cisplatin in cells.

**Results:**

The immunohistochemistry assay showed that adjuvant chemotherapy-treated NSCLC patients expressing EHD1 exhibited reduced OS compared with patients who did not express EHD1 (*P* = 0.01). Moreover, DNA microarrays indicated that the EHD1 gene was upregulated in CDDP- resistant NSCLC cells. The IC50 value of CDDP in cells that overexpressed EHD1 was 3.3-fold greater than that in the A549-control line, and the IC50 value of EHD1 knockdown cells was at least 5.2-fold lower than that of the control cells, as evidenced by a CCK-8 assay. We found that the percentage of early apoptotic cells was significantly decreased in A549-EHD1 cells, but the rates of early apoptosis were higher in the EHD1 knockdown cell line than in the A549/DDP control line, as indicated by a flow cytometry analysis. High-performance liquid chromatography (HPLC) showed that the total platinum level was lower in A549-EHD1 cells than in control cells, and the concentration of CDDP was higher in the EHD1 knockdown cells than in the A549/DDP control cells.

**Conclusion:**

We conclude that EHD1 is required for tumour growth and that it is a regulator of CDDP accumulation and cytotoxicity. The selective knockdown of EHD1 in tumours offers a strategy for enhancing the efficacy of CDDP.

**Electronic supplementary material:**

The online version of this article (doi:10.1186/s12885-016-2527-3) contains supplementary material, which is available to authorized users.

## Background

Lung cancer is one of the most devastating types of cancer and poses a serious threat to human life and health [1]. Specifically, it is the leading cause of cancer-related morbidity and mortality worldwide [2]. Non-small cell lung cancer (NSCLC) is the most common form of lung cancer and accounts for 80–85 % of all diagnosed lung cancers with a 5-year survival rate of 15 % [2]. Cisplatin (CDDP) is a component of standard treatment regimens for NSCLC [3], and adducts of CDDP with DNA induce apoptosis [4, 5]. However, many patients develop resistance during sequential cycles of treatment with CDDP, and this resistance undermines the efficacy of CDDP [6]. Drug mechanisms are complex and include decreased drug accumulation, increased drug efflux, altered oncogene expression, the activation of detoxification systems, impaired apoptosis and changes in the targets of the drug [7]. Recent studies suggest that many CDDP-resistant cells show decreased CDDP accumulation, and the identification of specific proteins for drug resistance should provide targets for therapy aimed at circumventing or decreasing CDDP resistance.

Cells internalize extracellular material, segments of the plasma membrane and cell surface receptors by endocytosis [8–10]. The C-terminal EPS15 homology (EH) domain (EHD) is a highly conserved family of proteins involved in endocytic trafficking [11]. This family consists of four highly homologous members in mammalian cells, EHD1-4 [12]. EHDs contain an ATP- binding motif, a central coiled-coil and a C-terminal EH domain that binds to proteins containing the tripeptide asparagin-proline-phenylalanine (NPF) [13]. EHD1 is the best characterized of the four EHD proteins [11] and has been demonstrated to play a role in regulating the recycling of receptors from the endocytic recycling compartment (ERC) to the plasma membrane [11]. EHD1 also plays a role in the transport of receptors from the early endosome (EE) to the ERC [11]. Moreover, EHD1 is also involved in retrograde transport from endosomes to the Golgi complex [11]. However, only a few studies have analysed the function of EHD1.

In this study, two independent cell lines that in which EHD1 was stably overexpressed or knocked down were established. The mechanism underlying EHD1-dependent CDDP resistance in NSCLC cells was investigated. Overall, our results suggest that EHD1 is a CDDP-resistant gene that suppresses DNA adduct-induced apoptosis by modulating intracellular CDDP concentrations. The present study sought to examine a novel therapeutic strategy to target CDDP-resistant lung cancer and provide theoretical evidence for the clinical application of this strategy.

## Methods

### Clinical tissue samples and IHC

The FFPE specimens of 59 patients with histologically confirmed NSCLC treated at the Harbin Medical University Cancer Hospital from 2006 to 2007 as well as their available clinical and follow-up information were examined in this study. All patients underwent surgical resection followed by three to four cycles of platinum-based adjuvant chemotherapy. This study was approved by the Institutional Review Board of the Harbin Medical University Cancer Hospital. All patients had to have provided written informed consent.

The sections were deparaffinized, autoclaved, treated with hydrogen peroxide solution, incubated with anti-EHD1 rabbit polyclonal antibody (Abcam) at a dilution of 1:50 overnight at 4 °C. A streptavidin–biotin complex system was used for staining. OS (overall survival) was calculated as the time to death from the date of diagnosis. Survival curves for OS were estimated with the Kaplan–Meier method and compared with the log-rank test [1, 9].

### Gene expression microarray

Fresh tumour tissues from 205 patients with NSCLC were obtained from the Harbin Medical University Cancer Hospital. Each patient had signed informed consent for tissue sample donation and medical record review before surgery. This study was approved by the Institutional Review Board at Harbin Medical University Cancer Hospital. The tissues were then stored in RPMI-1640 for the primary culture of NSCLC cells. None of the patients had received neoadjuvant chemotherapy or radiation therapy at the time of surgery. In total, 199 tissue samples were tested for chemosensitivity with an MTT (Sigma) assay. The samples with the highest sensitivity or resistance to all of the 5 drugs (NVB, GEM, DOC, TAL, CDDP) were selected. Total RNA was then extracted (TRIzol, Invitrogen), and the RNA purity was assessed with a BioAnalyser 2100 instrument (Agilent). Gene expression was profiled using a Human Genome U133 Plus2.0 Affymetrix array (Santa Clara) according to the manufacturer’s instructions. Total RNA was reverse-transcribed using an RNA amplification kit (Ambion), which was then hybridized, washed and stained before scanning the chips. The mean SD of replicate spots was calculated for each gene using the Acuity 4.0 software (Molecular Devices) [7].

### Cell lines and cell culture

The human lung adenocarcinoma cell line A549 was cultured in RPMI-1640 medium (Gibco), supplemented with 10 % foetal bovine serum (FBS, Gibco) and 1 % penicillin-streptomycin (HaiGene). The cells were maintained at 37 °C in a humidified atmosphere of 5 % CO_2_, and A549 cells were then selected for resistance to cisplatin. A series of cisplatin-resistant A549 populations (A549/DDP) were selected by stepwise increases in the cisplatin (Sigma) concentration from 0.3 μg/ml to 20 μ g/ml over a period of 8 months [14, 15]. EHD1 was then stably overexpressed or knocked down in tumorigenic cells or in CDDP-resistant cells using lentiviral expression vectors. The lentiviral expression vectors were purchased from HaiGene. The sequence of the EHD1-shRNA was 5′- CGCTTTCCTCAACAGGTTCATTTCAAGAGAATGAACCTGTTGAGGAAAGCG-3′. Lentiviral transfection was performed according to the methods described by the manufacturer (HaiGene). The cells were then cultured in the above culture medium containing Puromycin (Sigma) 0.5 μg/mL. Transfection efficiency was monitored by measuring the level of EHD1 mRNA using quantitative reverse transcription–polymerase chain reaction (RT– PCR) or immunofluorescence.

### RT-PCR assay

Total RNA was extracted from cultured cells using the OMEGA Kit following the manufacturer’s instructions. Total RNA was used to synthesize cDNA (Reverse Transcription Kit, Roche). The newly synthesized cDNA was then amplified by RT-PCR. Real-time PCR was performed on the Applied Biosystems 7500 Fast Real Time PCR System. The expression of indicated genes was assessed by PCR using the following primers: for EHD1 — 5-CCAAGGTTCACGCCTACATC-3 (forward), 5-TCTCCCAGGTTGTTCACCAG-3 (reverse); primers were purchased from Sangon Biotech. The mRNA expression level of EHD1 was calculated using the comparative threshold cycle (CT) method and normalized to the expression of GAPDH (Life Science).

### Immunofluorescence

Cells were cultured on cover slips for 24 h and then fixed with 4 % paraformaldehyde for 20 min and permeabilized with 0.2 % Triton X-100 for 10–15 min at room temperature. After washing with PBS, the cells were blocked with normal goat serum for 30 min. The cells were then incubated with antibodies (Proteintech) specific to EHD1 diluted at 1:50 overnight at 4 °C, followed by secondary antibody incubation with Alexa Fluor 488-labeled Goat Anti-Rabbit IgG (Beyotime) for 1 h and staining with DAPI (Beyotime) for 5 min at room temperature. The stained cells were mounted in antifade mounting medium (Beyotime) and visualized under a fluorescence microscope.

### Cell viability assay

The cells were seeded in 96-well plates (8,000 cells per well) and incubated overnight. After attachment, the cells were treated with various concentrations of CDDP for 24 and 48 h; cells treated with only RPMI-1640 medium served as controls. After cisplatin treatment, 10 μL of CCK-8 (Dojindo) was added to each well at 37 °C in the dark. After 3 h, the absorbance was measured at 450 nm using an Microplate Reader (BioTek). The IC50 values were determined using the IBM SPSS Statistics 19 software.

### Western blotting

Cells were harvested with trypsin, pelleted by centrifugation at 4 °C and disrupted in cell lysis buffer (Roche) on ice for 15 min. The protein concentration was determined with a BCA Protein Assay Kit (Thermo) as described in the manufacturer’s manual. Protein lysate (50 μg) was subjected to sodium dodecyl sulphate-polyacrylamide gel electrophoresis (SDS-PAGE) and electrophoretically transferred to a polyvinylidene difluoride membrane. After blotting, the membrane was incubated with a specific primary antibody (EHD1: Proteintech; GAPDH: ZSGB-BIO) at 4 °C overnight. After washing with TBST (TBS containing 0.05 % Tween 20), the membrane was incubated with secondary antibodies (1:10000; ZSGB-BIO) for 1 h at room temperature. Protein bands were visualized using a Super ECL Reagent (HaiGene), and the protein expression was quantified by densitometry.

### Flow cytometry

Cells were plated in 6-well plates and incubated overnight. After attachment, the cells were treated with various concentrations of CDDP for 24 h. For flow cytometry, cells were harvested and labelled using an Annexin V FITC/propidium iodide (PI) apoptosis detection kit (BD) according to the manufacturer’s instruction. At least 500,000 cells were analysed per sample. Apoptotic cells were quantified using a flow cytometer (BD FACSCalibur). Cells that were Annexin V+/PI- were considered to be in the early apoptotic stage.

### High-performance liquid chromatography

CDDP was dissolved in a 0.9 % (w/v) sodium chloride solution (1 mg/ml), which was used as the stock solution for HPLC analysis. Each standard solution for HPLC analysis was prepared by diluting the stock solution consecutively with 0.9 % (w/v) sodium chloride solution. The cells were treated with various concentrations of CDDP. The lysed cells samples were centrifuged at 4 °C for 15 min at 13000 rpm. The cell samples were added, mixed with Na_2_CO_3_ and NaOH (5 % DDTC, v/v), vortexed briefly, and incubated at 37 °C for 30 min. The cell samples were precipitated by the addition of ether-chloroform-isopropanol and centrifuged at 4 °C for 5 min at 10000 rpm; the supernatant was discarded. All samples were frozen and stored below -20 °C until analysis.

The Shimadzu 10AT HPLC apparatus was operated at a wavelength of 254 nm. Solutions were injected into an ODS Hypersil column (Agilent technologies) using an autosampler (Shimadzu) and eluted using two pumps. The mobile phase was methanol-water (80:20, v/v), which was supplied at a constant flow-rate of 1.0 ml/min. The chemicals used to prepare the mobile phase of the chromatographic system were of HPLC grade, and all other chemicals were of analytical grade. All other chemicals and reagents were purchased from BOSTER. A constant injection volume of 20 μL was used throughout the study, and the baseline was set to zero after the injection of the sample [16].

### Statistical analysis

The data were statistically analysed using Student’s *t*-test or the *χ*_2_ test with GraphPad Prism 6.0, and *P* < 0.05 was considered to indicate significant differences. All experiments were repeated three times, and representative data are shown. *P < 0.05, **P < 0.01, ***P < 0.001.

## Results

### EHD1 correlated with the poor clinical outcome of NSCLC

**Fig. 1:**
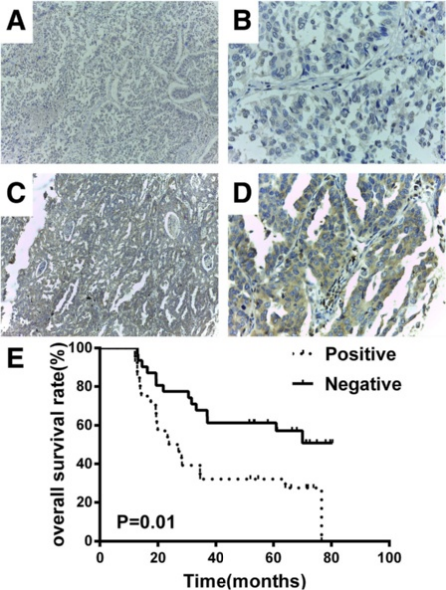
IHC analysis of EHD1 expression and correlation between EHD1 and survival in NSCLC tissues. EHD1-negative (**a**:x100; **b**x400:) and EHD1-positive (**c**:x100;**d**:x400) tumour samples are shown. Tissue sections were stained with EHD1-specific antibodies, and an immunohistochemical analysis was conducted as described in the Materials and Methods. **e**: OS in NSCLC patients receiving adjuvant chemotherapy. Patients receiving post-operational adjuvant chemotherapy with positive EHD1 expression exhibited reduced OS (*P* = 0.01)

To determine the roles of EHD1 in the clinical behaviour of NSCLC, we first used an IHC assay to profile EHD1 expression in FFPE specimens from 59 patients with NSCLC. EHD1 expression significantly differed by histological type (*P* = 0.001) (60.47 % of adenocarcinoma samples expressed EHD1, whereas 12.50 % of squamous cell carcinoma expressed EHD1) and sex (*P* = 0.006) (Table 1). EHD1 expression in NSCLC did not correlate with age, smoking status, differentiation, and stage (AJCC). EHD1 was expressed in 47.46 % NSCLC samples (Fig. 1, Table 1). Specifically, EHD1 was not expressed in CDDP-sensitive cases (Fig. 1a and b) but highly expressed in the NSCLC tissues of the CDDP-resistant cases (Fig. 1c and d). The OS of the adjuvant chemotherapy-treated patients with negative EHD1 expression was significantly longer than that of patients with positive EHD1 expression (*P* = 0.01) (Fig. 1e).

**Table 1:** Clinical characteristics of the NSCLC patients stratified by EHD1 level

Characteristic	Positive	Negative	*P*
Age (years)			
High (≥60)	9	14	0.306
Low (<60)	19	17	
Sex			
Male	13	25	0.006
Female	15	6	
Histology			
Adenocarcinoma	26	17	0.001
Squamous cell carcinoma	2	14	
Smoking status			
Smokers	15	24	0.053
Non smokers	13	7	
Differentiation			
Well/Moderate	13	12	0.549
Poor	15	19	
Stage (AJCC)			
I,II	19	26	0.149
III,IV	9	5

A gene expression microarray was used to analyse 5 samples exhibiting the highest resistance and 6 samples exhibiting the highest sensitivity to CDDP, and fold changes in gene expression were analysed by SAM. A fold-change in EHD1 expression of at least 1.2906 ± 0.07 was considered to be differential expression, but this difference was not significant (Table 2). Compared with the CDDP-sensitive group, EHD1 was upregulated in the chemotherapy-resistant group. These results suggested that the up-regulation of EHD1 might confer resistance to CDDP in NSCLC patients.

**Table 2:** Level of EHD1 gene upregulation in CDDP-resistant NSCLC cells assessed by DNA microarray analysis

Gene	Function	Fold change
EHD1	EH domain-containing protein	1.3419
EHD1	EH domain-containing protein	1.2393

### EHD1 overexpression and knockdown at both the protein and transcript levels using lentiviral expression vectors

**Fig. 2:**
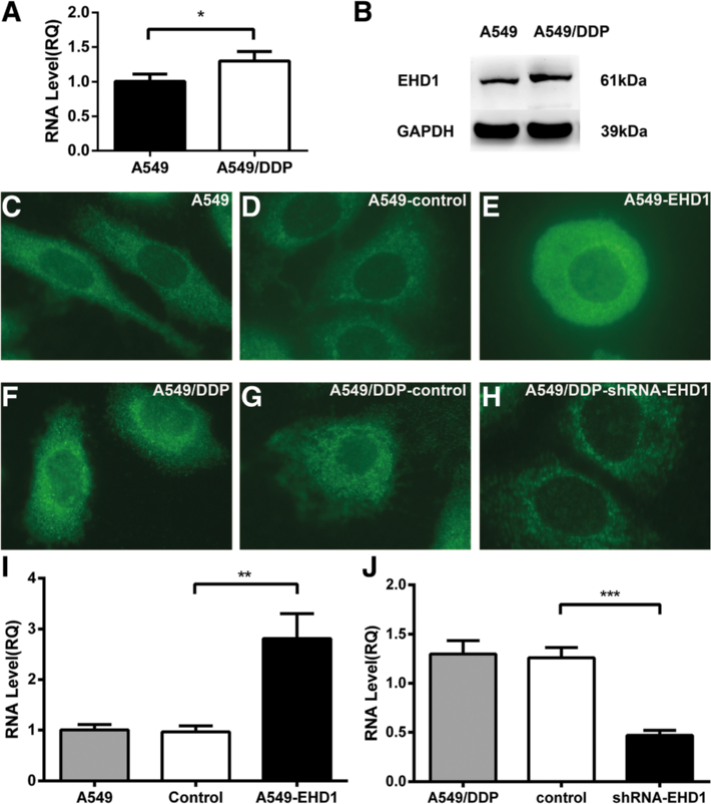
Establishment of EHD1-overexpressing and EHD1-knockdown NSCLC cells. EHD1 was confirmed at both the transcript and protein level in A549 and A549/DDP cell lines (**a** and **b**). The NSCLC cell line A549 was transfected with control or EHD1 overexpression (A549-EHD1) vector and analysed for EHD1 expression by immunofluorescence (**c-e**) and qRT–PCR (**i**). The CDDP-resistant cell line (A549/DDP) transfected with control or shRNA-EHD1 was analysed for EHD1 expression by immunofluorescence (**f-h**) and qRT–PCR (**j**)

EHD1 was confirmed at both the transcript and protein level in A549 and A549/DDP cell lines (Fig. 2a and b). The localization of EHD1 protein expression in NSCLC cell lines was examined by fluorescence microscopy in living cells. EHD1 was localized in the perinuclear region, yielding a ring-like pattern (Fig. 2c-h). Whereas a diffuse green fluorescence signal due to EHD1 was observed throughout the entire cell in A549-EHD1 cells, the overexpressed EHD1 was predominantly localized in the cytoplasm, forming a ring-like pattern at the nuclear periphery in cells that stably expressed EHD1 (Fig. 2e). EHD1 overexpression was confirmed at the transcript level (Fig. 2i). To evaluate the effect of EHD1 on cisplatin resistance, we knocked down EHD1 using shRNA. The A549/DDP cells were transfected with a shRNA-EHD1 and analysed by immunofluorescence microscopy. EHD1 expression detected by the EHD1 antibody consistently displayed a peri-nuclear staining pattern, but the EHD1 levels are significantly decreased in A549/DDP-shRNA-EHD1 cells (Fig. 2h). The shRNA sequences efficiently knocked down the expression of EHD1. Figure 2j shows the reduction in EHD1 mRNA in response to EHD1 shRNA measured by RT-PCR. More importantly, these findings are reminiscent of those in clinical samples, in which EHD1 expression was intense in the perinuclear area in the CDDP-resistant specimens (Fig. 1a-d).

### Overexpression of EHD1 confers cytoprotection against CDDP in NSCLC cells

**Fig. 3:**
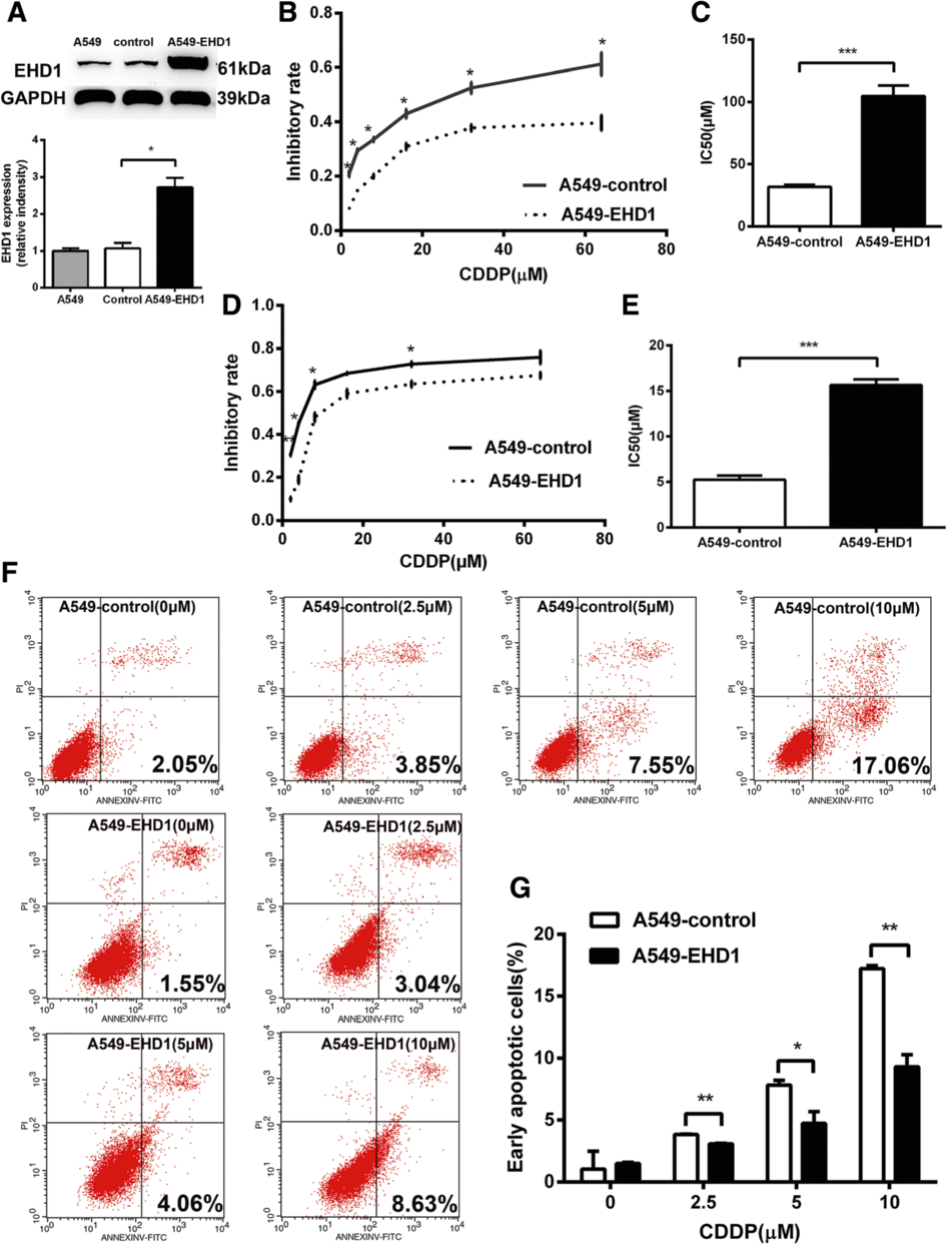
Overexpression of EHD1 confers resistance to CDDP in NSCLC cells. **a** Western blotting analysis to confirm the overexpression of EHD1. GAPDH was used as a control to verify the equal loading of protein. Relative protein levels of EHD1 were determined. **b** Inhibitory effect of CDDP was measured in NSCLC cells using the CCK-8 assay at 24 h. **c** The IC50 values for CDDP were determined in NSCLC cells at 24 h. **d** Inhibitory effect of CDDP was measured at 48 h. **e** The IC50 values for CDDP were determined at 48 h. **f** Annexin-V-FITC/PI staining was analysed by flow cytometry in NSCLC cells after treatment with CDDP for 24 h. **g** The histogram demonstrates the percentage of early apoptotic NSCLC cells in response to CDDP concentrations of 0, 2.5, 5 and 10 μM

Figure 3a shows that the EHD1 levels were significantly elevated in A549-EHD1 cells compared with the control, as evidenced by a western blot analysis. We next confirmed the effect of EHD1 on the efficacy of CDDP by stably overexpressing EHD1 in NSCLC cells. The indicated cell lines were treated with increasing concentrations (2, 4, 8, 16, 32 and 64 μM) of CDDP for 24 and 48 h before measuring inhibition. Cells that overexpressed EHD1 (A549-EHD1) exhibited higher survival rates than the control line in response to increasing concentrations of CDDP (Fig. 3b and d). The A549-EHD1 cells were 3.3-fold(24 h) and 3.0-fold(48 h) more resistant to cisplatin than the control cells, as determined by their IC50 values, respectively (Fig. 3c and e). To examine the effects of CDDP on NSCLC cell apoptosis, the cells were exposed to 0, 2.5, 5 or 10 μM CDDP for 24 h and analysed by flow cytometry. In A549-control cells, 2.5, 5 and 10 μM CDDP increased the population of apoptotic cells to 3.85, 7.55 and 17.06 %, respectively (Fig. 3f, g). In contrast, the proportion of early apoptotic cells only slightly increased in A549-EHD1 cells exposed to 2.5 or 5 μM CDDP (3.04 and 4.06 %, respectively), with the highest proportion (8.63 %) observed in the 10 μM CDDP treatment group (Fig. 3f, g). Overall, treatment with CDDP induced apoptosis in NSCLC cells but not in EHD1-overexpressing cells. Thus, the overexpression of EHD1 protected NSCLC cells from CDDP-induced cell death and increased survival.

### Knockdown of EHD1 enhances CDDP cytotoxicity in NSCLC cells

**Fig. 4:**
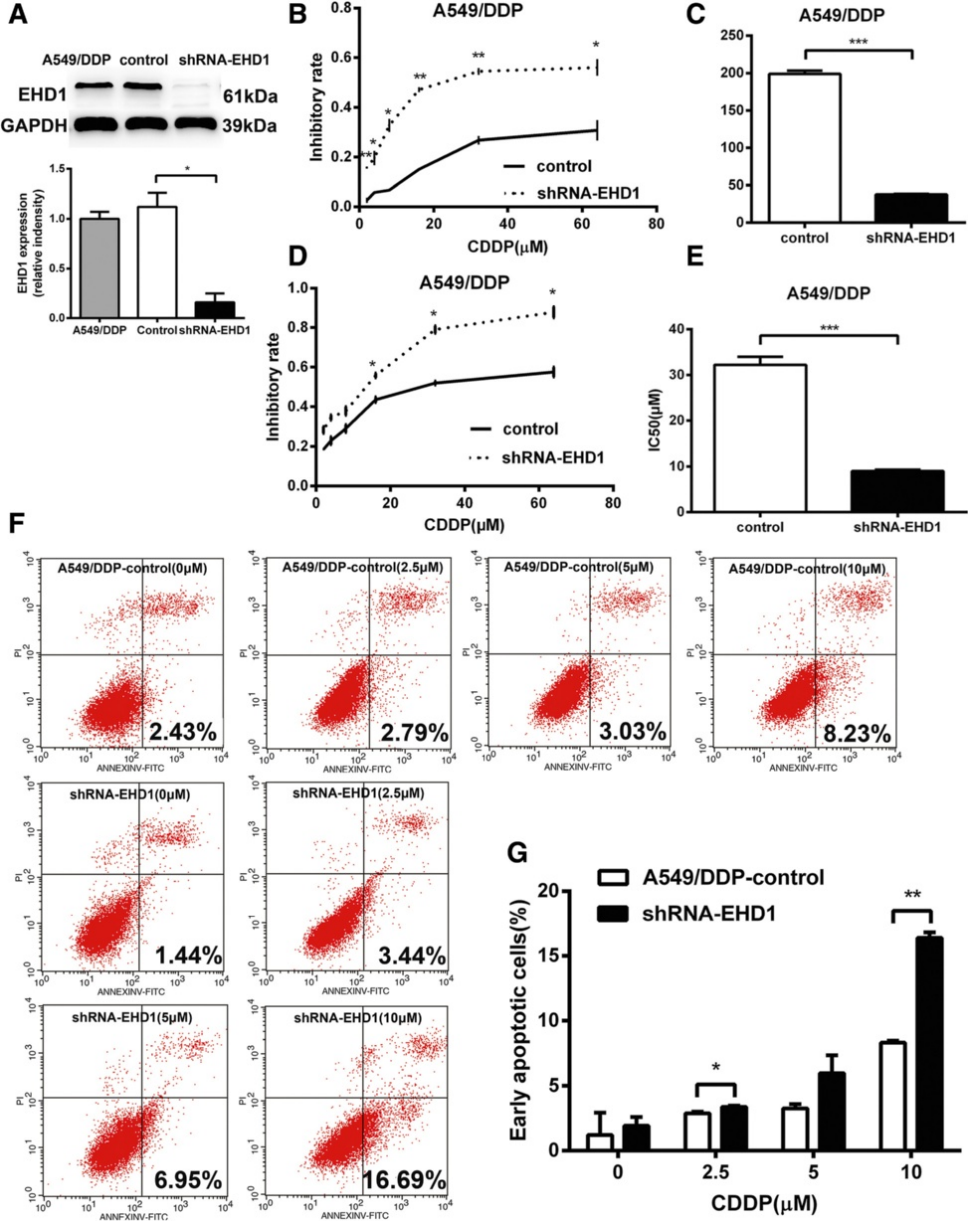
Knockdown of EHD1 increased sensitivity to CDDP in CDDP-resistant NSCLC cells. **a** Western blotting analysis of EHD1 levels. **b** Inhibitory effect of CDDP was measured in CDDP-resistant NSCLC cells using the CCK-8 assay at 24 h. **c** The IC50 values for CDDP were determined in CDDP-resistant NSCLC cells at 24 h. **d** Inhibitory effect of CDDP was measured at 48 h. **e** The IC50 values for CDDP were determined at 48 h. **f** Annexin-V-FITC/PI staining was analysed by flow cytometry in CDDP-resistant NSCLC cells after treatment with CDDP for 24 h. **g** The histogram demonstrates the percentage of early apoptotic in CDDP-resistant NSCLC cells in response to CDDP concentrations of 0, 2.5, 5 and 10 μM

Figure 4a shows that EHD1-shRNA considerably decreased EHD1 protein expression, as evidenced by a western blot analysis. We next tested the effect of CDDP inhibition on cell viability in CDDP-resistant NSCLC cells. The indicated cell lines were treated with increasing concentrations of cisplatin. After 24 and 48 h, CDDP inhibition markedly reduced cell viability in the CDDP-resistant cell line transfected with shRNA-EHD1 but did not markedly affect control cells (Fig. 4b and d). Moreover, the IC50 values for CDDP were at least 5.2-fold(24 h) and 3.6-fold(48 h) higher in A549 control cells than in the A549-shRNA-EHD1 line, respectively (Fig. 4c and e). We then examined differences in CDDP-induced apoptosis between EHD1 knockdown cells and control cells. Apoptosis in A549/DDP control and A549/DDP-shRNA-EHD1 cells treated with 0, 2.5, 5 and 10 μM CDDP for 24 h was assessed by flow cytometry. The proportion of early apoptotic cells only slightly increased in A549/DDP control cells exposed to 2.5, 5, 10 μM CDDP (2.79, 3.03 and 8.23 %, respectively), but this population significantly increased in EHD1 knockdown cells treated with 5 (6.95 %) and 10 μM (16.69 %) CDDP (Fig. 4f and g). The knockdown of EHD1 induced apoptosis in A549/DDP cells but not in control cells. Moreover, EHD1 knockdown significantly sensitized cells to cisplatin, suggesting that EHD1 knockdown could reverse CDDP resistance and enhance cytotoxicity in CDDP-resistant A549 cells.

### EHD1 correlated with the intracellular CDDP concentrations in NSCLC

**Fig. 5:**
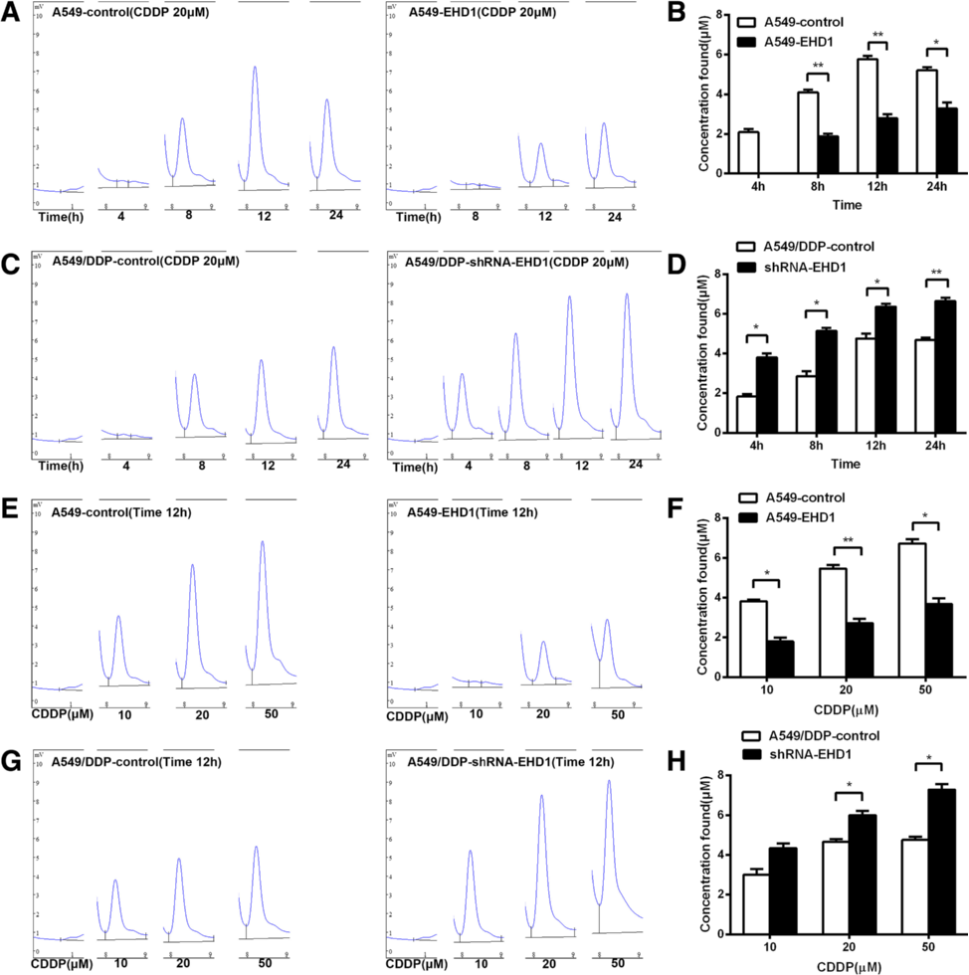
Determination of the CDDP concentrations in NSCLC cells treated with CDDP. **a** Chromatograms of extract (CDDP) from NSCLC cellular lysate (4, 8, 12, and 24 h). **b** Quantification of CDDP in lysate extracted from EHD1-overexpressing and control cell lines (4, 8, 12, and 24 h). **c** Chromatograms of extract (CDDP) from CDDP-resistant NSCLC cellular lysate (4, 8, 12, and 24 h). **d** Quantification of CDDP in lysate extracted from EHD1 knockdown and control cell lines (4, 8, 12, and 24 h). **e** Chromatograms of extract (CDDP) from NSCLC cellular lysate (10, 20, and 50 μM). **f** Quantification of CDDP in lysate extracted from EHD1-overexpressing and control cell lines (10, 20, 50 μM). **g** Chromatograms of extract (CDDP) from CDDP-resistant NSCLC cellular lysate (10, 20, and 50 μM). **h** Quantification of CDDP in lysate extracted from EHD1 knockdown and control cell lines (10, 20, and 50 μM)

The intracellular CDDP concentrations in NSCLC were investigated using HPLC. The concentration of CDDP in the cells was determined by measuring the total platinum concentration. The relationship between the cisplatin peak area and concentration was linear and characterized by a correlation coefficient of 0.998 (Additional file 1: Figure S1). CDDP was added to a blank cellular lysate, and the level of CDDP was monitored. The statistical analysis of the data revealed the recovery of CDDP from cellular lysates and the relative standard deviation of the assay (Additional file 2: Tables S1 and 2). CDDP was not detected in the blank cellular lysate samples. The indicated cell lines were exposed to 20 μM CDDP for 4, 8, 12 and 24 h and analysed in triplicate; Fig 5 shows one peak for cisplatin with a retention time of approximately 8.2 min. Figure 5a and c show chromatograms of CDDP in each cell line at the indicated time points. Figure 5b and d show the time courses of CDDP levels in each cell line. Specifically, the concentration of CDDP positively correlated with time. However, the increase in the CDDP concentrations of the control was significantly higher than that observed in EHD1-overexpressing A549 cells (Fig. 5a and b), and the increase in the CDDP concentration in EHD1 knockdown cells was significantly higher than that in A549/DDP control cells (Fig. 5c and d). Interestingly, the concentrations of CDDP extracted from the cell lines after 12 or 24 h did not significantly differ, although the 24 h time point exhibited higher concentrations of CDDP in each cell line than the 12 h time point (A549-EHD1,A549/DDP-control, A549/DDP-shRNA-EHD1). Thus, the indicated cell lines were exposed to 10, 20 and 50 μM CDDP for 12 h and analysed in triplicate. The results show differences in the CDDP concentrations between EHD1 overexpression and control cells. In A549-EHD1 cells, the total platinum level was lower than in control cells (Fig. 5e and f).

Similarly, high concentrations of CDDP were observed in EHD1 knockdown cells, but low concentrations of CDDP were observed in the control cells (Fig. 5g and h). These results indicate that EHD1 regulates the intracellular CDDP concentrations. The high concentration of CDDP in EHD1 knockdown cells could explain the clinical utility of CDDP in the treatment of NSCLC.

## Discussion

For patients with resectable stage IB to IIIA NSCLC, CDDP-based regimen is the most common choice for postoperative adjuvant therapy, but its clinical effectiveness is undermined by the inherent and acquired resistance of tumour cells to this drug. In this study, we observed that the expression of EHD1 was upregulated in cisplatin-resistant NSCLC cells, as indicated by DNA microarrays. Consequently, EHD1 expression predicted a poor OS in patients who received adjuvant chemotherapy. A previous study using DNA microarrays showed the EHD1 gene was upregulated at least 2-fold in HeLa-R1 cells or at least 4-fold in HeLa-R3 cells [7], and the upregulation of EHD1 was reported to be associated with cisplatin resistance. We favour the interpretation that platinum-based chemotherapy is more effective against EHD1-negative tumours. To further support these data, we established EHD1 overexpression and knockdown cell lines and then evaluated their response to CDDP.

Indeed, NSCLC cells that stably overexpressed EHD1 exhibited strong resistance to cisplatin. Consistent with these results, the IC50 value of CDDP in EHD1 knockdown cells was at least 5.2-fold(24 h) and 3.6-fold(48 h) lower than that in control cells, respectively. To understand the mechanism underlying the protective effect against CDDP-induced cytotoxicity, we examined the population of early apoptotic cells in CDDP-treated EHD1 overexpressing cells. The results suggest that the percentage of early apoptotic cells was significantly decreased in A549-EHD1 cells. We also found that the apoptosis index for A549/DDP cells remained low, but the rates of early apoptosis were higher in the EHD1 knockdown cell line than in the A549/DDP control line. These results suggest that knockdown of EHD1 sensitized platinum complex-resistant NSCLC cells to CDDP, but EHD1 overexpression might prevent CDDP-induced apoptotic death to protect NSCLC cells from the effects of CDDP. Specifically, our data suggest that the inhibition of EHD1 is an effective method to overcome CDDP resistance in NSCLC. These data have provided a novel biomarker and insight into the mechanism of chemoresistance in NSCLC.

Resistance to CDDP is a major obstacle in the clinical treatment of NSCLC. Multiple mechanisms are involved in the cytotoxic effects of CDDP, and resistance is commonly believed to be associated with the decreased intracellular accumulation of drugs [17]. The formation of DNA adducts by cisplatin can be limited by reduced drug accumulation in a tumour cell. By fluorescence microscopy, we found that EHD1 was expressed in the cytoplasm, forming a ring-like pattern at the nuclear periphery. Similarly, ERC is expressed near the nucleus. Thus, EHD1 co-localizes with ERC, suggesting its participation in cargo internalization [9]. Endocytosis can be classified into several types, including macropinocytosis, clathrin-mediated endocytosis and caveolin- mediated endocytosis [18]. A rapid recycling pathway transports cargo directly from the EE to the plasma membrane, whereas a second, slower pathway involves passage through the ERC [19]. The primary role of EHD1 appears to be in regulating the return of endocytosed cargo to the plasma membrane from ERC via a clathrin-dependent pathway [19]. However, the mechanisms are complex and not yet completely understood. EHD1 might associate with the cargo vesicles arriving from the early endosome on ERC membranes and link them to motor proteins [20]. The motor proteins would then drive these cargo vesicles for delivery back to the cell surface. Alternatively, EHD1, which exhibits ATPase activity, may promote the budding of vesicles from tubules that emanate from the ERC, thus facilitating vesicular transport to the cell surface [20]. Endocytic recycling has long been regarded as a trafficking route, and this process is important in the control of multiple signalling pathways and biological functions [21]. Resistance to cisplatin is likely due to the up-regulation of EHD1, which increases the trafficking of CDDP from the ERC to the plasma membrane and limits the ability of cisplatin to form DNA adducts in the nucleus. Similarly, the recycling of internalized CDDP to the cell surface is decreased in EHD1-depleted cells.

In this study, we sought to investigate the effect of EHD1 on CDDP accumulation. The HPLC method was successfully applied to in vitro studies and enabled us to quantify CDDP not only in the NaCl medium but also in the cellular lysates using only 0.02 mL of a sample. Our results clearly demonstrate that the intracellular CDDP concentrations in EHD1-overexpressing cells were lower than those in control cells. The cellular accumulation of CDDP, as measured by HPLC, was not significantly reduced in CDDP- resistant cells. However, the knockdown of EHD1 increased the intracellular accumulation of CDDP, which may effectively prevent drug release from cells and be related to the reduced excretion of CDDP. This findings suggests that EHD1 significantly affects the cellular accumulation of CDDP and consequently is an important determinant of the cytotoxicity of CDDP. The use of this HPLC system can unambiguously measure the level of CDDP in cells. This technique will be suitable for the quantitative determination of CDDP in cancer cells showing resistance and requires minimal sample preparation. Thus, this method is easily automated and will be useful for studies of drug therapies.

## Conclusion

These observations indicate that the overexpression of EHD1 reduced the accumulation of CDDP in the cell, significantly inhibiting adduct-induced apoptosis and contributing to drug resistance in NSCLC cells. The knockdown of EHD1 sensitizes cells to chemotherapy and reverses CDDP resistance in lung cancer cells via the accumulation of CDDP inside cells. Overall, these findings indicate that EHD1 is a tumour resistance-associated protein. Therefore, EHD1-targeted therapy may improve the treatment of CDDP-resistant tumours to increase survival in patients with lung cancer.

## Abbreviations

CDDP, cisplatin; EE, early endosome; EHD1, Eps15 homology domain - containing protein 1; ERC, endocytic recycling compartment; FFPE, formalin-fixed paraffin embedded; HPLC, High-performance liquid chromatography; IHC, immunohistochemical; NPF, tripeptide asparagin-proline-phenylalanine; NSCLC, non-small cell lung cancer; OS, overall survival; qRT-PCR, quantitative reverse transcription–polymerase chain reaction; SD, standard deviation

## Additional files

Additional file 1: The relationship between the cisplatin peak area and concentration was linear and characterized by a correlation coefficient of 0.998. (JPG 200 kb) Additional file 2: Intra-day precision and Inter-day precision. CDDP was added to a blank cellular lysate, and the level of CDDP was monitored. The statistical analysis of the data revealed the recovery of CDDP from cellular lysates and the relative standard deviation of the assay. (PDF 52 kb)
