# Supplementary figures and images for: EHD1 confers resistance to cisplatin in non-small cell lung cancer by regulating intracellular cisplatin concentrations
Source: BMC Cancer. 2016 Jul 13;16:470. doi: 10.1186/s12885-016-2527-3 (PMC4944258; doi:10.1186/s12885-016-2527-3)

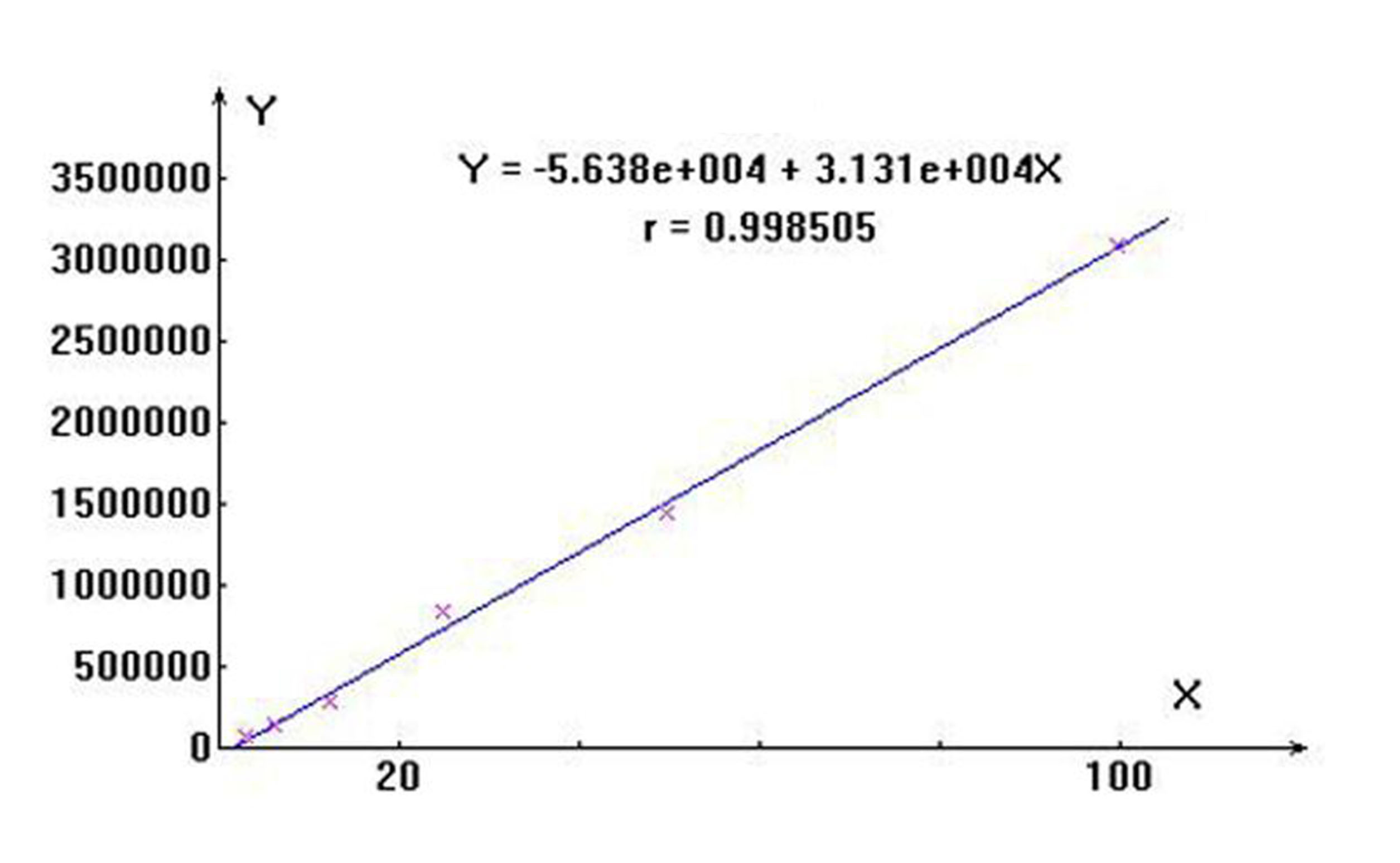

Supplement: Additional file 1: — The relationship between the cisplatin peak area and concentration was linear and characterized by a correlation coefficient of 0.998. (JPG 200 kb) [file 12885_2016_2527_MOESM1_ESM.jpg]
