# Supplementary material for: EHD1 confers resistance to cisplatin in non-small cell lung cancer by regulating intracellular cisplatin concentrations
Source: BMC Cancer. 2016 Jul 13;16:470. doi: 10.1186/s12885-016-2527-3 (PMC4944258; doi:10.1186/s12885-016-2527-3)
Supplement: Additional file 2: — Intra-day precision and Inter-day precision. CDDP was added to a blank cellular lysate, and the level of CDDP was monitored. The statistical analysis of the data revealed the recovery of CDDP from cellular lysates and the relative standard deviation of the assay. (PDF 52 kb) [file 12885_2016_2527_MOESM2_ESM.pdf]

Supplementary Table 1. Intra-day precision

CDDP added (μm/L)	Platinum concentration (μm/L)	Recovery	Average recovery	RSD
2	1.95	97.50%	99.00%	0.026
	1.99	99.50%		
	2	100.00%		
6	6.08	101.33%	101.11%	0.023
	6.08	101.33%		
	6.04	100.67%		
10	9.5	95.00%	95.37%	0.042
	9.52	95.20%		
	9.59	95.90%		

Supplementary Table 2. Inter-day precision

CDDP added (μm/L)	Platinum concentration (μm/L)	Recovery	Average recovery	RSD
2	1.96	98.00%	99.05%	0.020
	1.98	99.00%		
	2	100.00%		
6	5.95	99.17%	99.56%	0.049
	5.94	99.00%		
	6.03	100.50%		
10	9.76	97.60%	98.61%	0.044
	9.75	97.50%		
	9.68	96.80%		
